# Effect of Topographic Data Accuracy on Watershed Management

**DOI:** 10.3390/ijerph16214245

**Published:** 2019-11-01

**Authors:** Ismail Fathy, Hany Abd-Elhamid, Martina Zelenakova, Daniela Kaposztasova

**Affiliations:** 1 Department of Water and Water Structures Engineering, Faculty of Engineering, Zagazig University, Zagazig 44519, Egypt; ismailfathy_eng@yahoo.com (I.F.); hany_farhat2003@yahoo.com (H.A.-E.); 2 Civil Engineering Department, College of Engineering, Shaqra University, Duwadimi 11911, Saudi Arabia; 3 Department of Environmental Engineering, Faculty of Civil Engineering, Technical University of Košice, Košice 04200, Slovakia; 4 Department of Architectural Engineering, Faculty of Civil Engineering, Technical University of Košice, Košice 04200, Slovakia

**Keywords:** watershed management, DEM, SRTM, ASTER GDEM, GTOPO30

## Abstract

A digital elevation model (DEM) is a digital model or 3D representation of a terrain’s surface. There are many methods to create DEM such as LiDAR, stereo photogrammetry and topographic maps. DEMs are very important for many applications such as extracting terrain parameters for geomorphology and modeling water flow for hydrology or mass movement. A number of websites are available to provide DEM such as SRTM, GTOPO30 and ASTER GDEM but their accuracy differs from one to another and also selecting a small DEM size (high resolution) gives accurate information, but the analysis takes long time. This paper aims to analyze the impact of using different available DEMs on watershed geomorphological properties on order to provide guidelines for users to select the most suitable DEM that obtain an accurate analysis in less time. Three programs; watershed modeling systems: WMS, Global Mapper and Google Earth were used in this study. Three case studies were studied to check the accuracy of these models and select the most accurate one for application. Satellite images downloaded from Google Earth were used as a guide reference for the comparison due to their accuracy and high resolution. The results indicated that the SRTM model was more accurate (95%) for all case studies according to our comparison between its delineation and satellite images. ASTER GDEM is the second most accurate model with an accuracy of 87%, the GTOPO30’s accuracy is 80%.

## 1. Introduction

Analyses of climatology, hydrology, geology, agriculture and geomorphology mainly depend on the topography (Earth surface features). The accuracy of the topographic data affects the outputs and analysis process. There are various global datasets of topography that have produced Digital Elevation Models (DEMs) with accuracy from 10 arc-minutes, used in modeling of more detailed Earth surface processes, to 30 arc-seconds which are used in hydrology, pedology, or small-scale geomorphology. Digitization or photogrammetry to produce high-resolution DEMs is expensive in terms of time and money [1].

Different open sources can be used to get DEMs such as the Thermal Emission and Reflection Radiometer (ASTER) Global Digital Elevation Model (GDEM) which known as ASTER GDEM, the Shuttle Radar Topography Mission (SRTM) and Global 30-Arc-Second Elevation Data Set (GTOPO30). These sources can produce a homogeneous DEM with a three arc-seconds (around 90 m) grid mesh. A number of factors affect the precision of satellite-based DEMs such as systematic errors, errors that occur during data collection and unknown errors that are geographically dependent on the terrain conditions that cannot be avoided. Checking the performance and validation of the DEM sources is essential. In order to do that, ground control points must be surveyed that taken as a reference points to validate the downloaded DEMs [2].

Data collection is considered the main factor affecting the accuracy of satellite-based DEMs [3]. The insufficiency in orientation of images with photogrammetrically determined elevation values produces errors which are called systematic errors [4]. In addition, terrain conditions create random errors which cannot be avoided such as geographically dependence [5]. Furthermore, grid spacing and interpolation techniques produce DEMs errors that were identified by Mukherjee et al. [4]. Rabus et al. [6] studied the SRTM DEM accuracy in different regions. The results of the study indicated that the absolute vertical accuracy is about 16 m and the horizontal accuracy is around 20 m. Rodriguez et al. [7] expressed a different opinion, that the vertical accuracy of around 6.2 m and the horizontal accuracy 9.0 m. Kellndorfer et al., [8] proved that the DEMs errors increase in forested areas due to the presence of trees since the C-band beam used by SRTM only partially penetrates into the canopy.

The Shuttle Radar Topography Mission (SRTM) dataset for some regions was released by the National Aeronautics and Space Administration (NASA) in 2003 with accuracy 1 arc-second for the United States and 3 arc-second resolutions for the globe. This procedure represented a qualitative leap for hydrological research and facilitated practical applications in this field for small scale and large scale areas [1]. A number of researchers have validated DEMs using reference points (ground control points) and the accuracy was determined by measuring the percentage of errors using statistical accuracy indicators such as root mean square error (RMSE) or standard deviation [9].

A number of free global topographic data are available such as GTOPO30 in 1996, Shuttle Radar Topography Mission (SRTM) in 2000, Advanced Space borne Thermal Emission and Reflection Radiometer (ASTER), Global Digital Elevation Model (GDEM) in 2009. SRTM has an absolute vertical height accuracy of about 16 m (at 90% confidence) [2]. In 2009 ASTER GDEM version-1 was released through a joint collaboration between NASA and the Ministry of Economy, Trade, and Industry (METI) of Japan. Stereo-pair images collected by the ASTER instrument on board Terra were used to generate topographic data with 30 m spatial resolution. ASTER GDEM version-2 (GDEM2) was released in 2011 with several improvements such as the use of an additional 260,000 stereo-pairs from 2000 to 2010, improved coverage and reduced occurrence of artifacts and a refined production algorithm [10].

A comparison between open source DEMs using Differential Global Positioning System (DGPS) points was presented by Patel et al. [11]. The main goals were to develop a proper surface interpolation created by different interpolation techniques such as Inverse Distance Weighting (IDW), Global Polynomial Interpolation (GPI), Radial Basis Functions (RBF), Ordinary Kriging (OK), Universal Kriging (UK) and Local Polynomial Interpolation (LPI) which are usually used in geomorphology investigation. The result showed that the Cartosat-1 (30 m) data product is preferable over ASTER (30 m) and SRTM (90 m) because it generated a low RMSE of 3.49 m without applying any interpolation technique.

The statistical and physical properties of a DEM of the Brazilian territory was verified using extracted topographic data. Statistical indicators were calculated and the results indicated that the quality of SRTM data was enhanced by using topographic survey data [9]. Wong et al. [12] studied the precision of ASTER GDEM and SRTM with high precision topographic data of Light Detection and Ranging (LiDAR) acquired using a Riegl LMS-Q560 sensor. The study was conducted in a tropical montane forest area of approximately 3600 hectares in Borneo (Malaysia). They resampled both SRTM (90 m resolution) and ASTER GDEM (30 m resolution) with a bilinear interpolation and cubic convolution methods to one, two and five-meter pixel resolutions. Gesch et al. [13] evaluated the ASTER (GDEM v-2) data over the boundaries of the United States. The validation process was done using 18,000 control points (surveyed ground points). The results indicated that the difference errors equaled 8.68 m compared to 9.34 m for GDEM v-1. Thomas et al. [14] compared the sensitivity of various spaceborne DEMs such as Global Multi-resolution Terrain Elevation Data 2010 (GMTED), ASTER and SRTM. The comparison was done using survey data that had a contour interval of 20 m. The Pambar River Basin and Muthirapuzha River Basin were compared to estimate the differences. The results showed that spaceborne DEMs are better than SRTM and ASTER and are harmonious with the survey data.

Recently a number of researchers have presented comparisons between different DEMs from different point of view. Tri Dev et al. [15] matched two global DEMs: Shuttle Radar Topography Mission Global 30 m (SRTM30) and Advanced Land Observing Satellite World 3D 30 m (AW3D30) with reference resampled LiDAR DEM 30 m data in a test area located around Chuncheon, Korea. The statistics of each DEM such as their basin, difference, profiles, stream orders, and slope are used for assessment. The results indicate that AW3D30 and SRTM30 match very well but are inconsistent in the test area compared to the LiDAR30 data. In addition, SRTM30 shows less differences from LiDAR30 compared to the AW3D30 DEM. Hector and Cameron [16] presented multi-scale relief model (MSRM), which is a novel algorithm for the graphical interpretation of landforms using DSMs. The import technique relies in the ability to abstract landform morphology from both high and low resolution DSMs. An important advantage of that technique is that it allows the use of worldwide medium resolution models, such as ASTER GDEM, and SRTM.

Alganci et al. [17] compared DSMs obtained from a diversity of satellite radars and examined their accuracy and performance, including freely available DSM data such as Advanced Land Observing Satellite (ALOS) 30 m resolution, ASTER GDEM 30 m and SRTM 30 m. Moreover, 3 m and 1 m resolution DSMs were formed from tri-stereo images from the SPOT 6 and Pleiades high-resolution (PHR) 1A satellites, respectively. Elevation survey points (control points) were used to perform accuracy assessments. The accuracies of different DSMs were tested using a different number of checkpoints determined by different methods. The results indicated that SPOT and PHR are the best DEMs for the study.

This study used three case studies to apply three models to get the best model that can fully represent the study area. The main objective of this research was to compare the stream delineations created from digital elevation models that can be download from various available sites such as SRTM, ASTER GDEM and GTOPO30. Satellite images downloaded from the Google Earth program were used as a guide reference for this comparison due to their accuracy and high resolution.

## 2. Materials and Methods

In this study a number of watershed management models were applied to different case studies to highlight the effect of using the available DEMs from different sites on watershed geomorphological properties and delineation.

### 2.1. Watershed Management Models

A number of watershed management models are used in this study such as Global Mapper, WMS and the Google Earth program. First, the Global Mapper is used to get DEMs from different sites and used to manipulate the DEM files that will be used as input to WMS. second, WMS is used for stream delineation and to determine the watershed boundaries and properties. Finally, the Google Earth program is used to download geo-images that are used as a reference to compare the WMS results. The descriptions of these programs are given in the following sections.

#### 2.1.1. Watershed Modeling System (WMS)

WMS is a widespread graphical model that deals with hydraulics and watershed hydrology applications. WMS offers the possibility to determine watershed properties (slope, area and maximum flow distance) depending on the DEM data of the case study. Also, the runoff discharge and storage water volume can be calculated using losses methods and feeding storm data [18]. Table 1 illustrates the main features of the WMS model [18].

#### 2.1.2. Global Mapper

Global Mapper, created by Blue Marble Geographic (Hallowell, ME, USA) runs under Microsoft Windows. Global Mapper is a geographic information system (GIS) software package. Global Mapper handles both raster elevation data and vectors and provides conversion, viewing, and other general GIS features [19]. Global Mapper’s functionalities can be summarized as distance and area calculations, a digitizer tool for adding custom features, spectral analysis, feathering, raster blending, data attribute querying.

#### 2.1.3. Google Earth Program

The Google Earth program is a computer program that provides a 3D representation of Earth based on satellite pictures. The program has general awareness of geospatial technologies and applications. The program maps form a 3D globe permitting users to see houses and cities from numerous angles. Imagery resolution ranges about from 15 m to 15 cm. Users may use Google Earth to supplement their own data, making them available through many sources, such as forums or blogs. Google Earth supports managing 3D geospatial data through Keyhole Markup Language (KML) [20].

### 2.2. Case Studies

**Figure 1:**
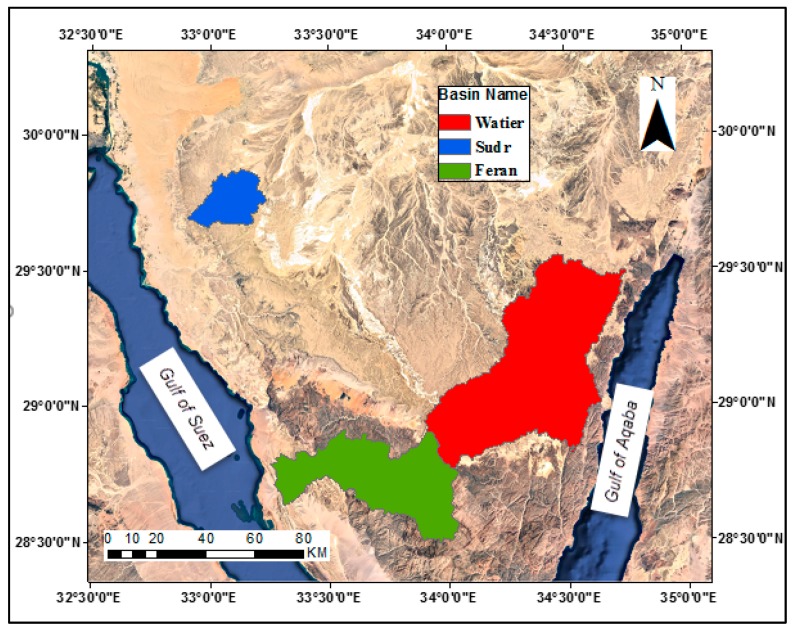
Location map of the study areas.

Three case studies (Wadi Sudr, Wadi Feran and Wadi Watier) at different locations in Sinai Peninsula, Egypt are selected to compare the performance and accuracy of digital elevation models DEMs. Streams delineation are created from the available DEMs and compared with the available satellite images. Figure 1 shows location map of study areas. The figure shows that Wadi Sudr is located at Middle West border of Sinai Peninsula, Wadi Feran is located at the west border and Wadi Watier is located at the east border of Sinai Peninsula.

#### 2.2.1. Wadi Sudr

**Figure 2:**
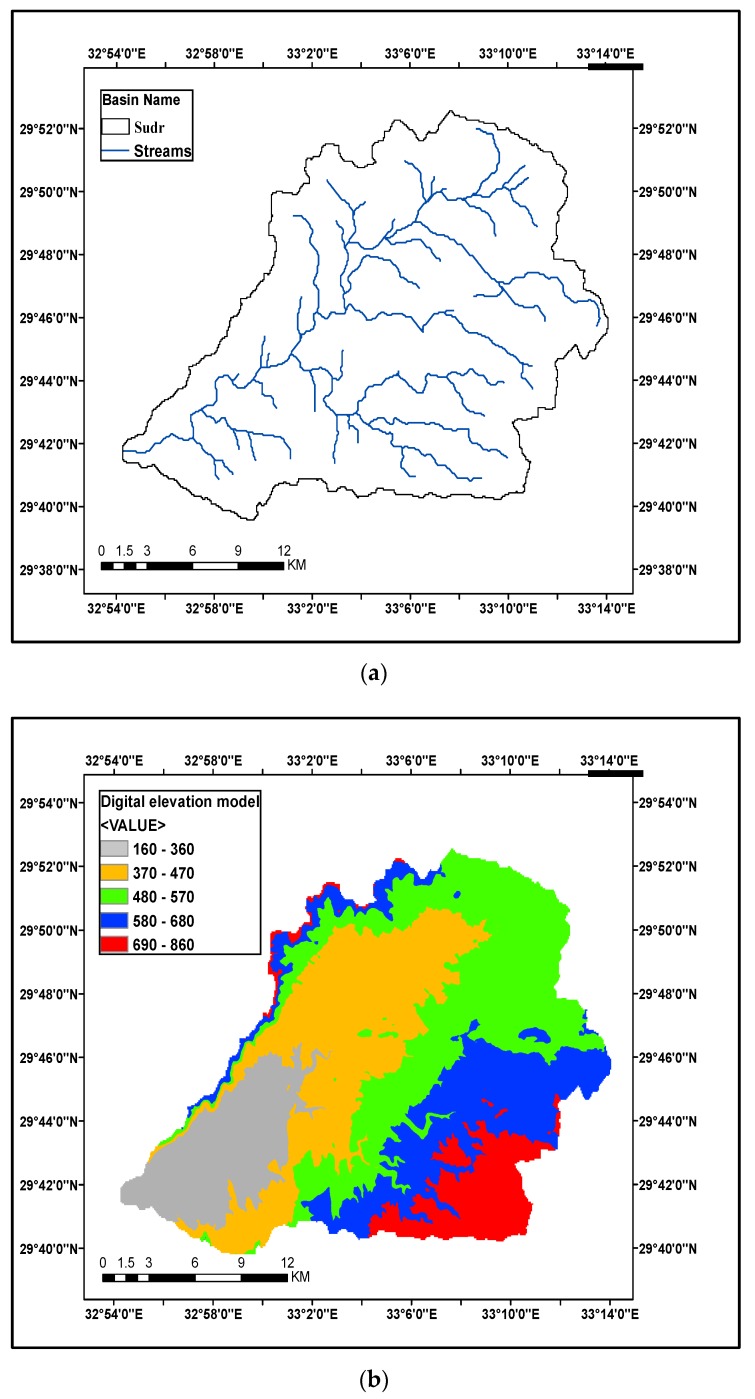

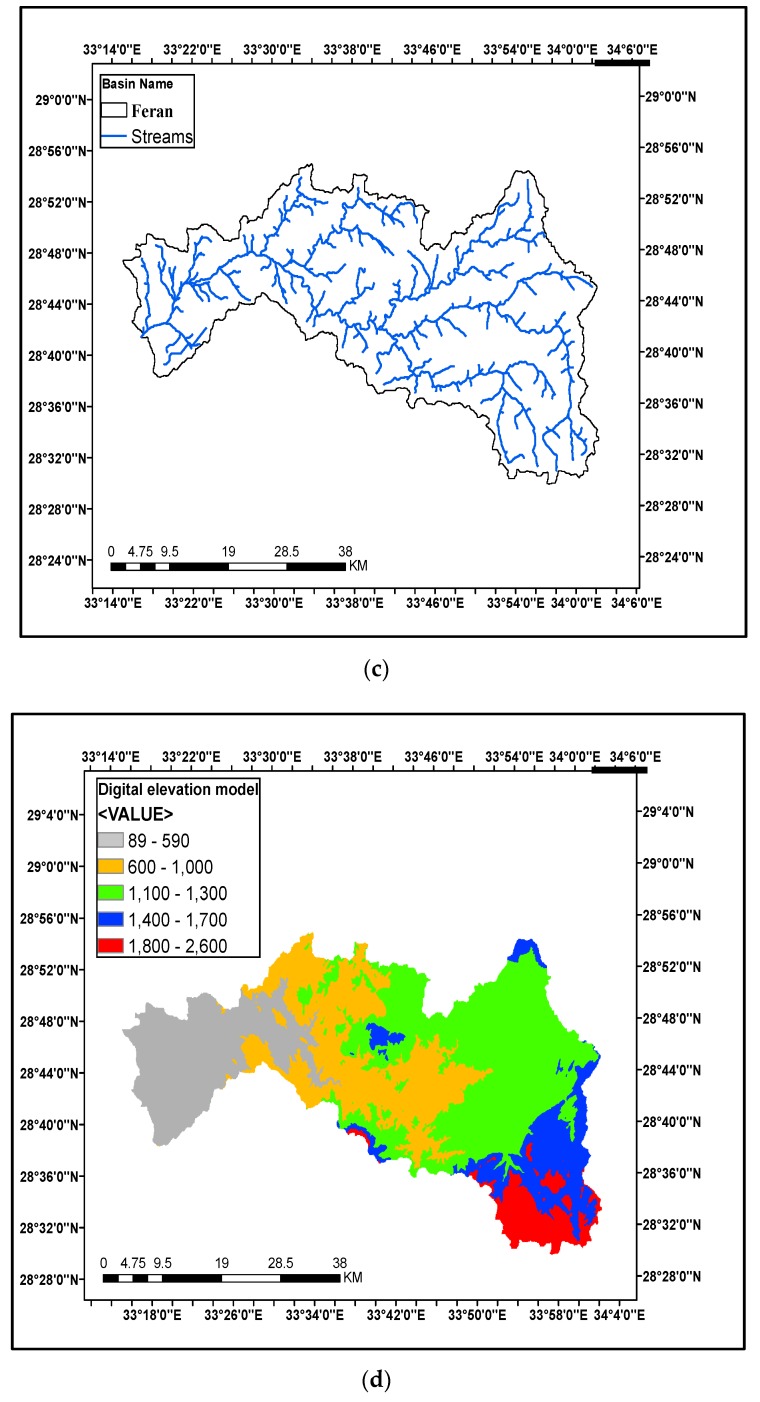

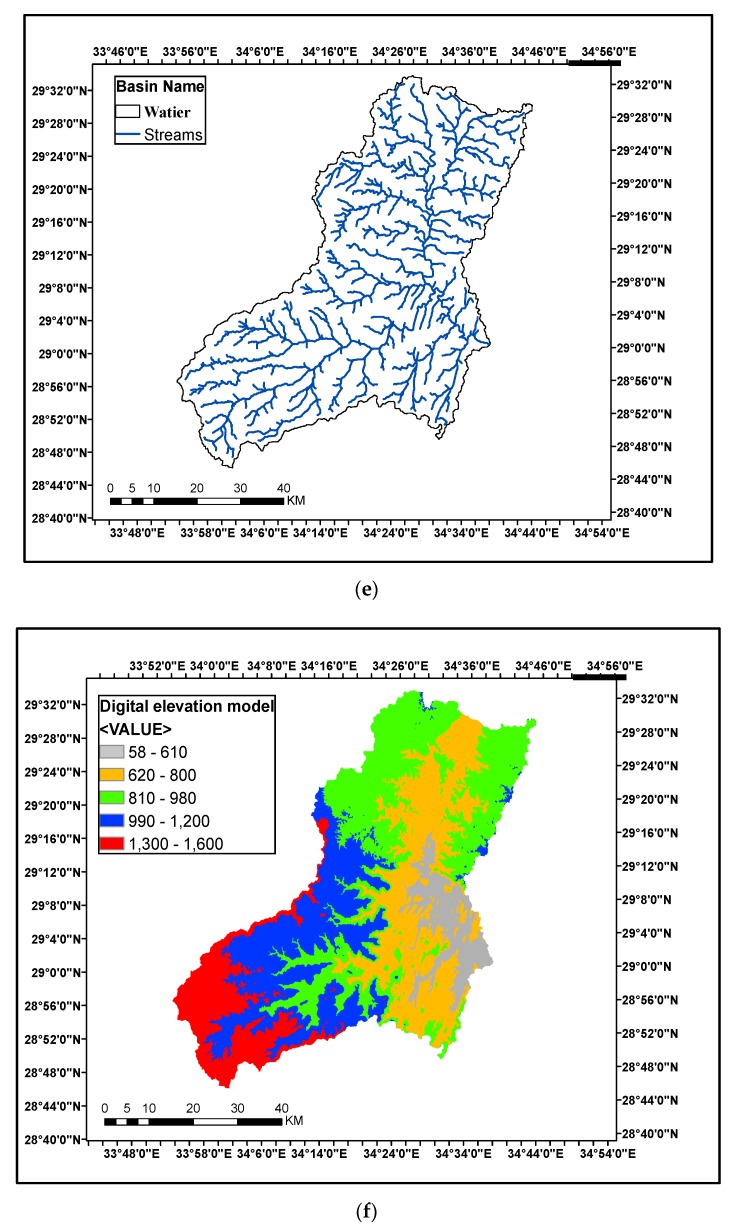
Stream delineation and digital elevation model of the cases studies: (**a**) Stream delineation and boundaries of Wadi Sudr; (**b**) Digital elevation model of Wadi Sudr; (**c**) Stream delineation and boundaries of Wadi Feran; (**d**) Digital elevation model of Wadi Feran; (**e**) Stream delineation and boundaries of Wadi Watier; (**f**) Digital elevation model of Wadi Watier.

Wadi Sudr is one of south-west Sinai wadis, which is located between latitudes 29°35′ and 29°55′, and longitudes 32°40′ and 33°20′ as shown in Figure 2a. Wadi Sudr covers a total area of about 600 km^2^. It drains water directly to the Gulf of Suez at Sudr town. Rainfall and runoff for this wadi have been measured by the Water Resources Research Institute (WRRI) since 1989 [21]. The data extracted from the digital elevation model indicates varying levels of the Earth’s surface, which ranges from 800 m above sea level, at Mount Umm Hammat, to the lowest level at the delta of the valley that flows in the Gulf of Suez as seen in Figure 2b.

#### 2.2.2. Wadi Feran

Wadi Feran is one of the valleys of South Sinai Governorate, which estuary ends in the Gulf of Suez. The first tributaries of the valley originate from Mount Catherine. The stream delineation and boundaries of Wadi Feran can be seen in Figure 2c. In addition, this valley is connected to the central Sinai and the Gulf of Suez, where it passes through the Catherine-Tunnel. The valley is exposed to frequent floods annually. Data from digital elevation model indicates varying elevation levels, ranging from 2000 m above sea level and reaching below 100 m at the exit of the valley near the Gulf of Suez as shown in Figure 2d.

#### 2.2.3. Wadi Watier

Watier Valley is one of the most important valleys that flow into the Gulf of Aqaba and ends the mouth of this valley at the city of Nuweiba, which is considered one of the most important economic centers in the Sinai Peninsula. The importance of Nuweiba city is due to the presence of the post of Nuweiba, which is considered one of the most important axes of intraregional trade among Arab countries. The stream delineation and boundaries of Wadi Watier can be seen in Figure 2e. Data from the digital elevation model indicates varying levels of the Earth’s surface, from 1500 m above sea level and reaching its lowest level at the delta of the valley that flows into the Gulf of Aqaba, as shown in Figure 2f.

## 3. Results

**Figure 3:**
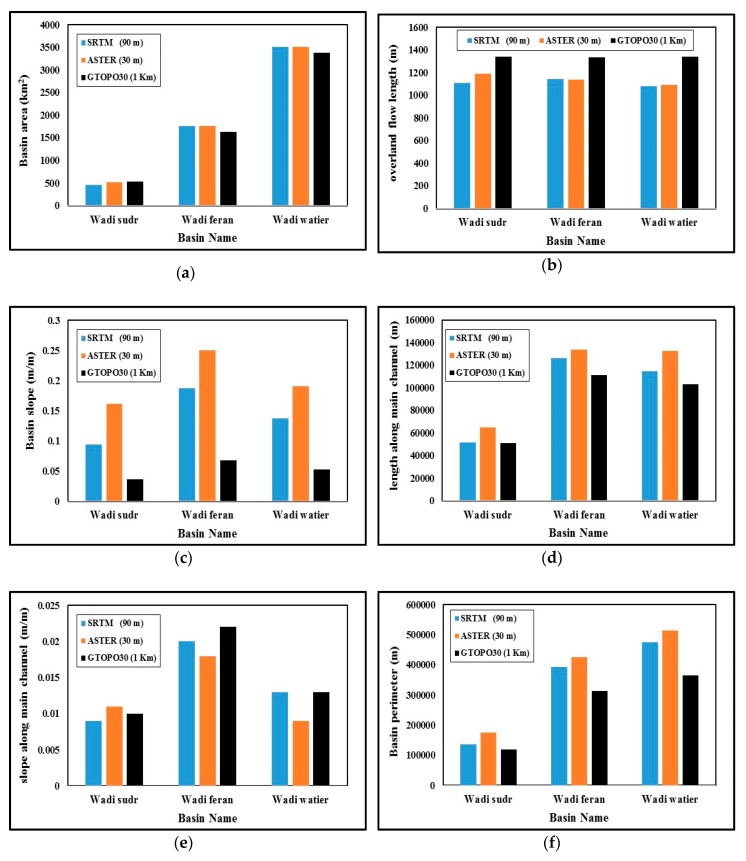
Comparison between geomorphological properties for the case studies: (**a**) Basin areas; (**b**) Basin overland flow lengths; (**c**) Basin slopes; (**d**) Basin lengths along main channel; (**e**) Basin slopes along main channel; (**f**) Basin perimeters.

The comparison between results of different DEMs for three cases studies is discussed in details in this section. The geometric properties developed from the available DEMs; SRTM, ASTER GDEM and GTOPO30 using watershed modeling system (WMS) for the three case studies; Wadi Sudr, Wadi Feran and Wadi Watier are shown in Table 2. The comparison between geometric properties (basin area, average overland flow length, basin slope, basin length along main channel, basin slope along main channel, basin perimeter and shape factor) is shown in Figure 3a–f.

Figure 3a shows a comparison between basin areas in the three case studies. For Wadi Sudr it is noticed that approximate values of basin areas calculation with disparity of 13% between SRTM and ASTER models and a disparity 16% between SRTM and GTOPO30 models. Also, for Wadi Feran the percentage of disparity for calculating area equal 0.30% between SRTM and ASTER models and 7.70% between SRTM and GTOPO30 models. In addition, for Wadi Watier the percentage of disparity for calculating are equal 0.15% between SRTM and ASTER models and 3.67% between SRTM and GTOPO30 models. This mean that a small gap for calculating areas, so calculating runoff volume during design detention basins or storage basins can be made using any of these models.

The length of overland flow is essential for calculating the time of concentrate which affects the peak discharge so determining this parameter is very important. As shown in Figure 3b the gap between SRTM and ASTER models is very small for the three cases and ranged between 0.42–7.25%. However, the gap between SRTM and GTOPO30 models ranged from 16.95–24%.

Basin slope affects the infiltration rate and time of peak discharge so the comparison of models output were made to verify this parameter as shown in Figure 3c. The gap between SRTM and ASTER models ranged between 34–71%, while on the other hand the gap between the SRTM and GTOPO30 models ranged from 61–63%. There is a large gap between the results of the three models as seen from the Figure 3c so site survey of small area is recommended or control ground point should be taken to calibrate the DEM file.

The length along the main channel is very important for flood routing calculations. The values of this parameter for the three models are shown in Figure 3d. The gap between SRTM and ASTER models ranged between 5.60–25.50%, while on the other hand the gap between SRTM and GTOPO30 models ranged from 1–11.90%. The gap between the three models is small.

The basin slope along the main channel is also important during flood routing and time of peak discharge calculation. The values of this parameter can be seen in Figure 3e, which shows the gap between SRTM and ASTER models ranged between 10–30.77%, whereas the gap between SRTM and GTOPO30 models ranged from 0–11.0%.

The basin perimeter is very important for calculation of the total water volume. The values of this parameter are shown in Figure 3f, where the gap between the SRTM and ASTER models ranged from 8–28%, while on the other hand the gap between the SRTM and GTOPO30 models ranged from 12–23%.

The basin shape factor is very important for determining drainage basin morphometrics. The differences between the results of the three models are very small. The gap between the SRTM and ASTER models ranged between 0–12%, while the gap between the SRTM and GTOPO30 models ranged from 0.6–13.0%.

**Figure 4:**
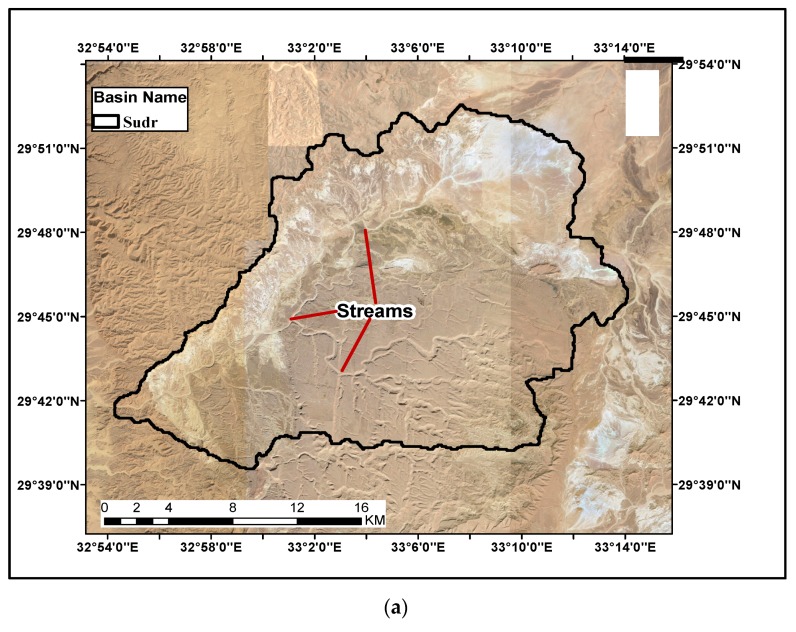

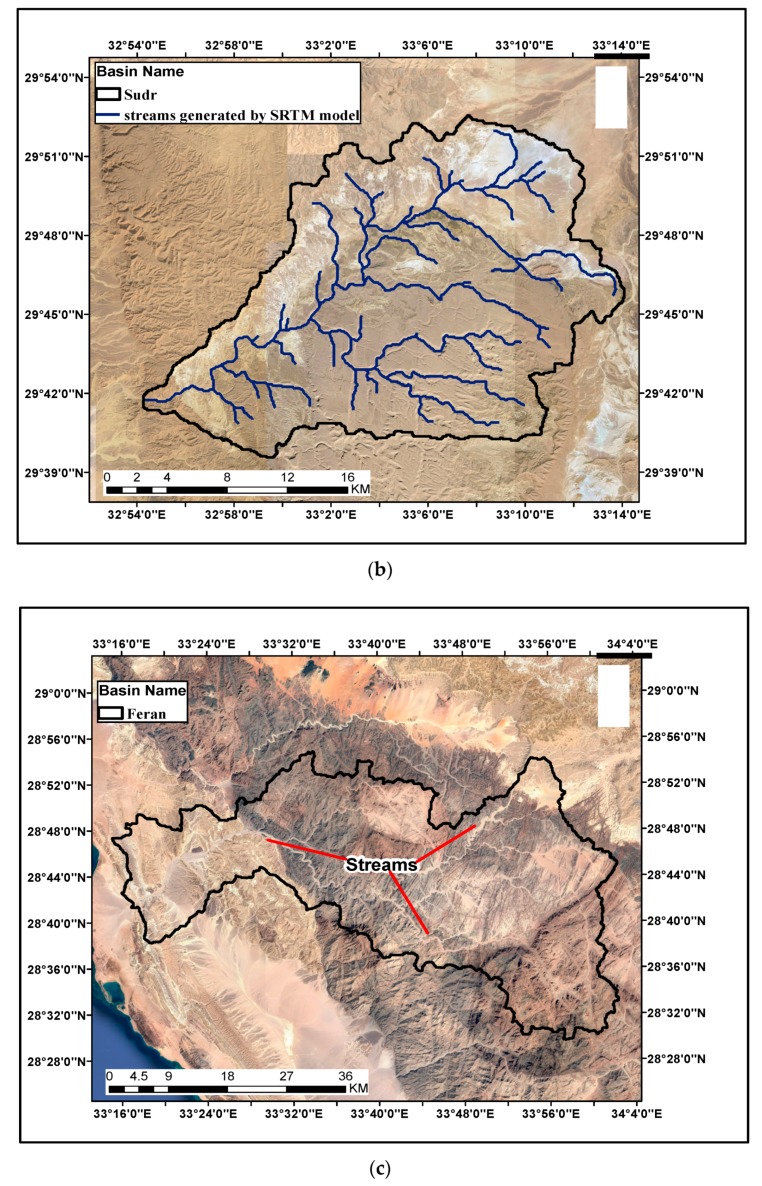

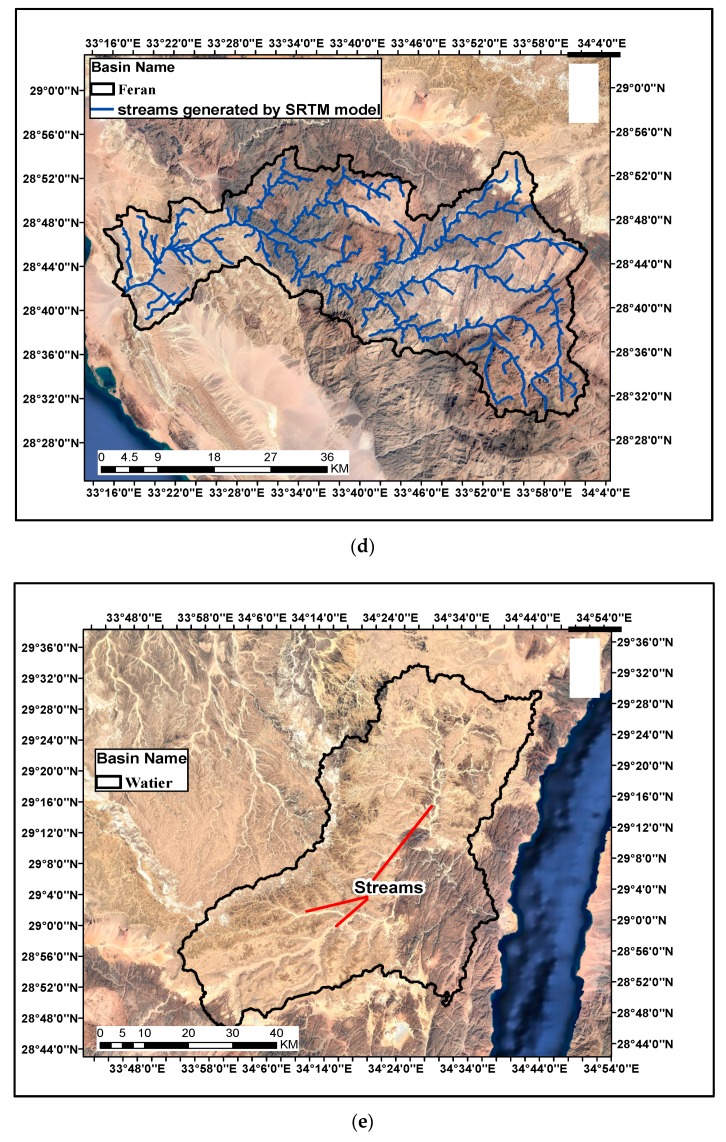

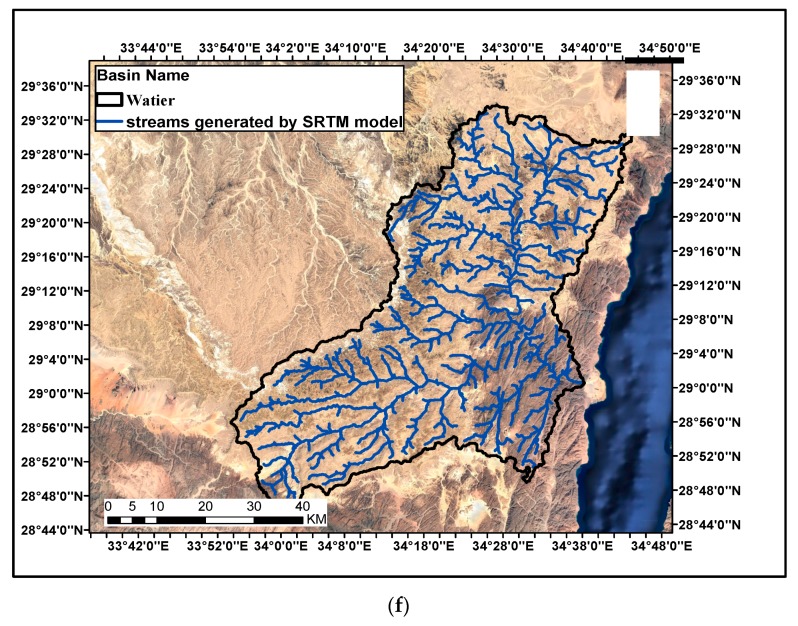
Comparison of streams generated by SRTM and satellite image for the case studies: (**a**) Satellite image by Google earth for Wadi Sudr; (**b**) Streams generated by SRTM for Wadi Sudr; (**c**) Satellite image by Google earth for Wadi Feran; (**d**) Streams generated by SRTM for Wadi Feran; (**e**) Satellite image by Google earth for Wadi Watier; (**f**) Streams generated by SRTM for Wadi Watier.

The reference index (satellite images) for the three case studies (Wadi sudr, Wadi Feran and Wadi Watier) are generated as shown in Figure 4a,c,e, respectively. These figures are downloadeded from Google Earth. The main streams for the three cases are represented in the figures. A comparison between satellite images and the stream delineation created from the SRTM model is shown in Figure 4b,d,f. The results indicated a good match between the delineation from SRTM and the satellite images for the three cases. The gap between the streams is measured and the percentage of error is calculated, which equals to 1%. Tis indicates that the SRTM model is suitable for the delineation process and determining the location of flood protection measures.

**Figure 5:**
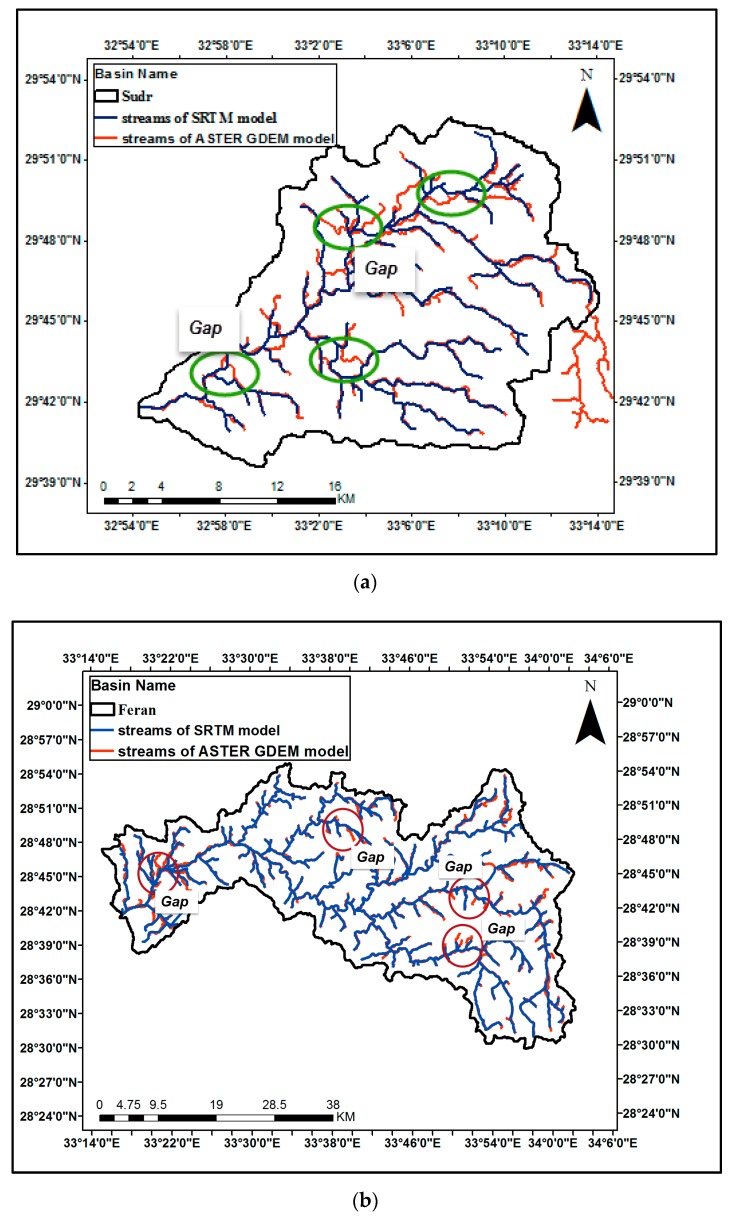

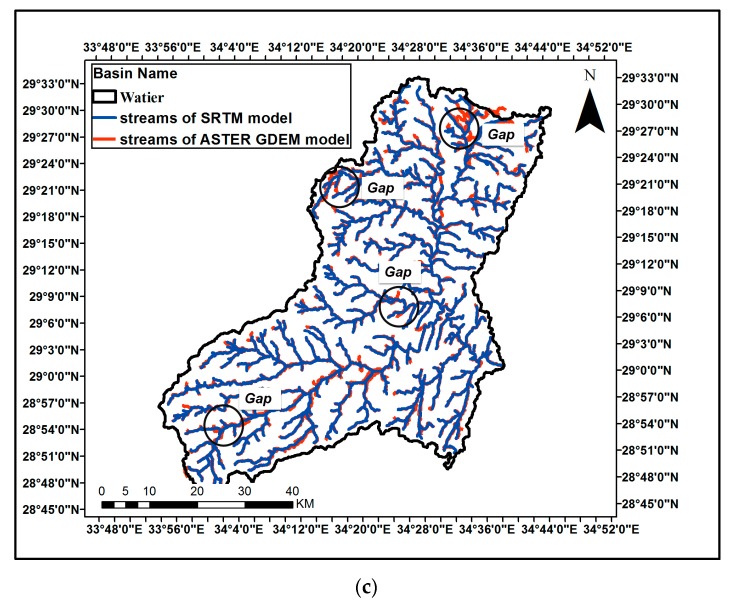
Comparison of streams generated by SRTM and ASTER GDEM for the case studies: (**a**) Streams generated by SRTM and ASTER GDEM for Wadi Sudr; (**b**) Streams generated by SRTM and ASTER GDEM for Wadi Feran; (**c**) Streams generates by SRTM and ASTER GDEM for Wadi Watier.

**Figure 6:**
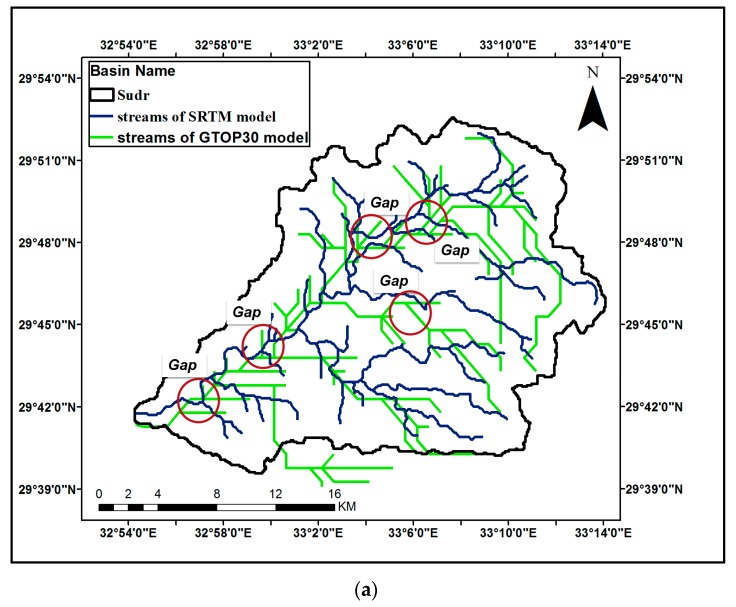

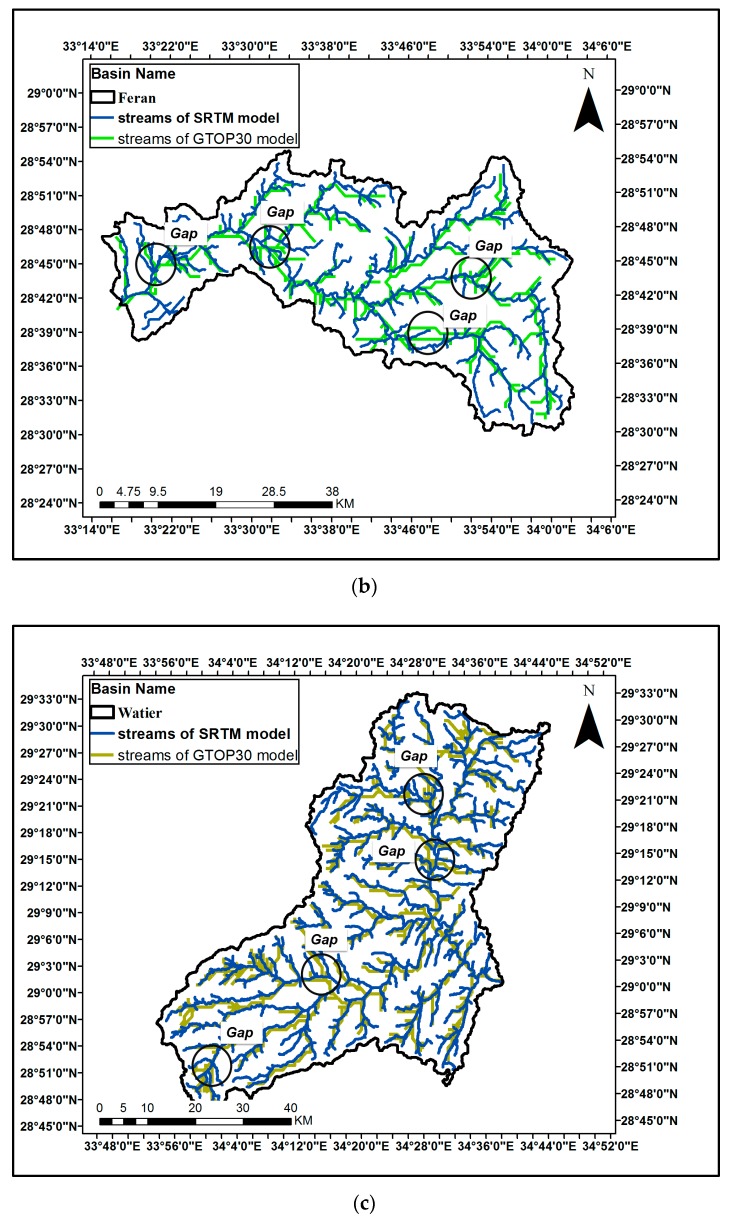
Comparison of streams generated by SRTM and GTOP30 for the case studies: (**a**) Streams generated by SRTM and GTOP30 for Wadi Suder; (**b**) Streams generated by SRTM and GTOP30 for Wadi Feran; (**c**) Streams generated by SRTM and GTOP30 for Wadi Watier.

Comparison of the streams generated by SRTM and ASTER GDEM is done for the three case studies as shown in Figure 5a–c. Some gaps of about 100 to 300 m are noted between the stream delineations of SRTM and ASTER GDEM which may affect the selection of the precise location of flood protection structures. Comparison of the streams generated by the SRTM and GTOPO30 models was also done for all case studies as shown in Figure 6a–c. Some gaps within about 700 to 2000 m between the delineations of the SRTM and GTOPO30 models are noticed that could affect the design of flood protection measures.

**Figure 7:**
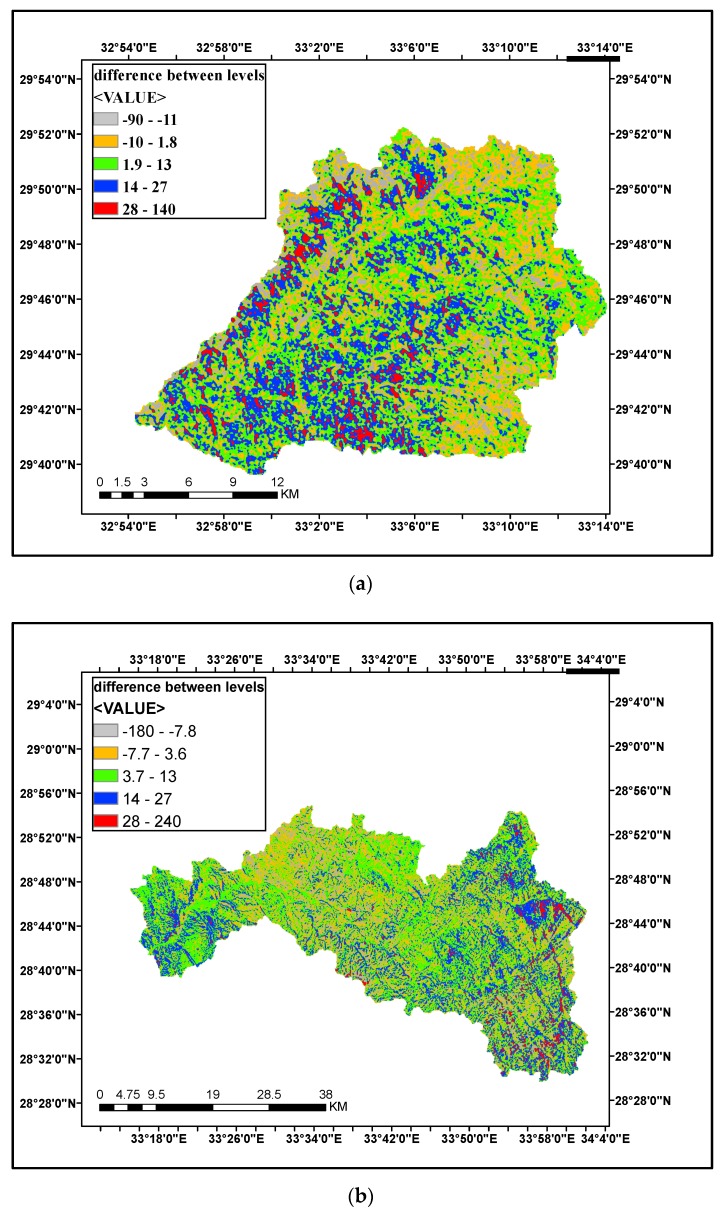

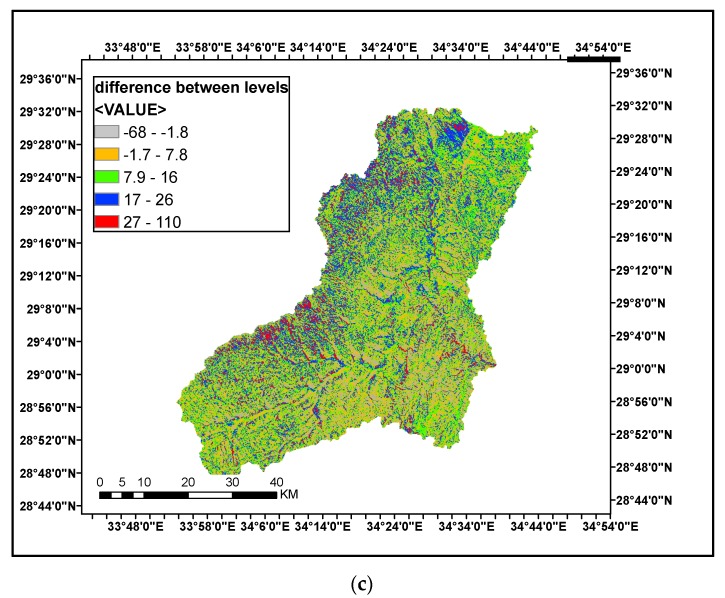
Comparison of surface of SRTM and ASTER (vertical levels) for the case studies: (**a**) Surface of SRTM and ASTER (vertical levels) for Wadi Sudr; (**b**) Surface of SRTM and ASTER (vertical levels) for Wadi Feran; (**c**) Surface of SRTM and ASTER (vertical levels) for Wadi Watier.

**Figure 8:**
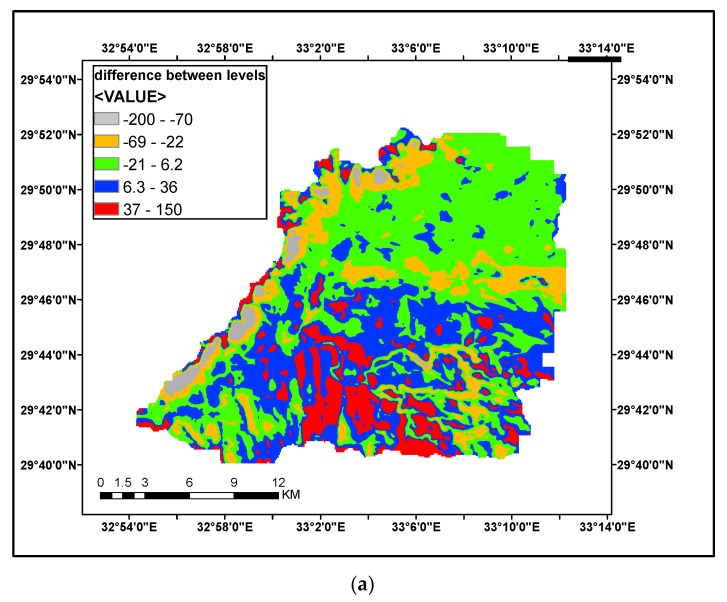

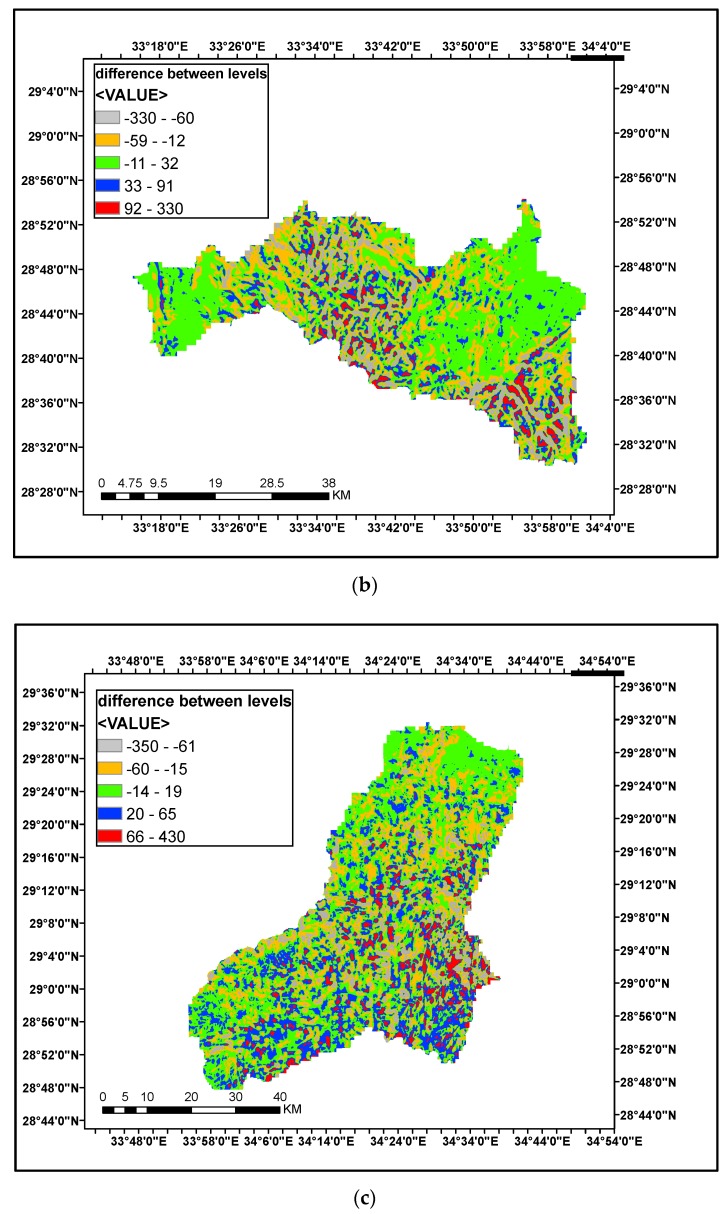
Comparison of surface of SRTM and GTOPO30 (vertical levels) for the case studies: (**a**) Surface of SRTM and GTOPO30 (vertical levels) for Wadi Sudr; (**b**) Surface of SRTM and GTOPO30 (vertical levels) for Wadi Feran; (**c**) Surface of SRTM and GTOPO30 (vertical levels) for Wadi Watier.

Surface levels homogeneity and different between models elevation levels were checked and comparison between levels was done as shown in Figure 7 and Figure 8. A small difference between SRTM and ASTER GDEM with a range of 50 m and a large difference between SRTM and GTOPO30 with a range of 200 m can be observed in Figure 7b.

A comparison between SRTM and ASTER GDEM was done (represented by the difference between ground levels) as shown in Figure 7a–c. It is noticed that the minimum difference occurring at Wadi Wateir is 68 m and the biggest values equals 241 m at Wadi Feran. 

## 4. Discussion

Comparing the results for different DEMs models that were downloaded from the available sites (SRTM, GTOPO30 and ASTER GDEM) showed that the SRTM model gave the lowest error when compared with satellite imagery, the second is ASTER GDEM and the lowest accuracy was produced by the GTOPO30 model. This study was applied for three wadies at different locations of Sinai Peninsula, Egypt. This study recommends the application of the SRTM model for computing different watershed management geometric properties and stream delineations due to its accuracy.

## 5. Conclusions

This paper presents a detailed comparison between the stream delineations created from different digital elevation models that are downloaded from various available sites such as SRTM, ASTER GDEM and GTOPO30. Satellite images downloaded from Google Earth are used as a guide reference for this comparison due to their accuracy and high resolution. The geometric properties for the three case studies were calculated and the results indicated that disparity ranged from 0.20 to 13% between the SRTM and ASTER models and from 7.7 to 16% between the SRTM and GTOPO30 models. The degrees of homogeneity of the variation of surface level were calculated and the results indicated a small difference between SRTM and ASTER GDEM on the order of 50 m and a large difference between SRTM and GTOPO30, with a range of 200 m. This study recommends the use of SRTM for different watershed areas to calculate geometric properties and delineations. It is known that using high accuracy DEMs increases the accuracy of the results but is time consuming. The current study showed that calculation of area and shape factor of basins does not require high resolution DEM, as the difference between the different DEM results was very small. Using low resolution DEMs for such a purpose could save time. However, for other basin properties high resolution DEMs are required. The current study recommends comparison between different softwares for watershed properties.

## Figures and Tables

**Table 1:** The main feature properties of the WMS model [18].

Model	WMS	
General	Reference	Environmental Modeling Research Laboratory of Brigham Young University
	Type	Watershed
	Scale	All sizes
	Interface	WINDOWS
Inputs	Precipitation	Frequency storm, user-defined, hyetograph, gridded precipitation, SCS
	Losses	Green-Ampt, SCS, gridded deficit constant, initial and constant
Convert equations	Surface Runoff	Kinematic wave, SCS unit hydrograph, clark unit hydrograph, user-specified unit hydrograph and Snyder unit hydrograph
Output		Peak discharge and runoff hydrograph

**Table 2:** The geometric properties of the three wadies using different DEMS.

Items	Wadi Sudr			Wadi Feran			Wadi Watier		
	SRTM (90 m)	ASTER (30 m)	GTOPO30 (1 Km)	SRTM (90 m)	ASTER (30 m)	GTOPO30 (1 Km)	SRTM (90 m)	ASTER (30 m)	GTOPO30 (1 Km)
Basin area Km^2^	460	521	533	1767	1762	1631	3512	3517	3383
Average overland flow length (m)	1109	1189	1341	1143	1138	1336	1082	1094	1341
Basin slope (m/m)	0.094	0.161	0.036	0.187	0.250	0.068	0.137	0.191	0.053
Basin length along main channel (m)	51,499	64,673	50,973	126,529	133,607	111,439	114,460	132,760	103,180
Basin slope along main channel (m/m)	0.009	0.011	0.010	0.020	0.018	0.022	0.013	0.009	0.013
Basin perimeter (m)	137,100	176,110	119,580	394,350	426,170	314,430	475,600	515,060	365,060
Shape factor	2.43	2.72	2.11	3.24	3.26	3.46	1.51	1.51	1.50
